# JOURNEY OF INNOVATION: ENGINEERING A BETTER LIFE

**DOI:** 10.1080/19498241.2024.2445971

**Published:** 2025-02-04

**Authors:** Linda Hosler, Rory A. Cooper

**Affiliations:** a United States Patent and Trademark Office; b Human Engineering Research Laboratories, VA Pittsburgh Healthcare System and University of Pittsburgh, Pittsburgh, PA, USA; c School of Health and Rehabilitation Sciences, University of Pittsburgh, Pittsburgh, PA, USA; d Swanson School of Engineering, University of Pittsburgh, Pittsburgh, PA, USA

## INTERVIEW

### RORY COOPER:

When I was first injured, I was told by the doctors that, you know, “you’d probably have a 10-year lifespan . . . ” And I could’ve just said, well then I’ll just hang out and do nothing and wait for my time to come instead of trying to do as much with my life as I could . . .

### LINDA HOSLER:

And so began a journey for Rory Cooper that made him one of the world’s leading experts in human mobility and accessibility engineering, as well as an advocate for veterans and the disabled.

I’m Linda Hosler from the United States Patent and Trademark Office. Professor Rory Cooper is an avid athlete whose life and career took a sharp turn at age 20. Stationed in Germany with the U.S. Army, he was hit by a bus while riding his bike, leaving him paralyzed from the waist down.

Cooper was determined to put the pieces of his life back together. Almost immediately, he began pursuing steps to become an engineer – subsequently completing a doctorate in the field of electrical and computer engineering. Building on his childhood passion of tinkering, he began to direct all of his efforts to making life more accessible for individuals with mobility impairments.

**Figure 1.**
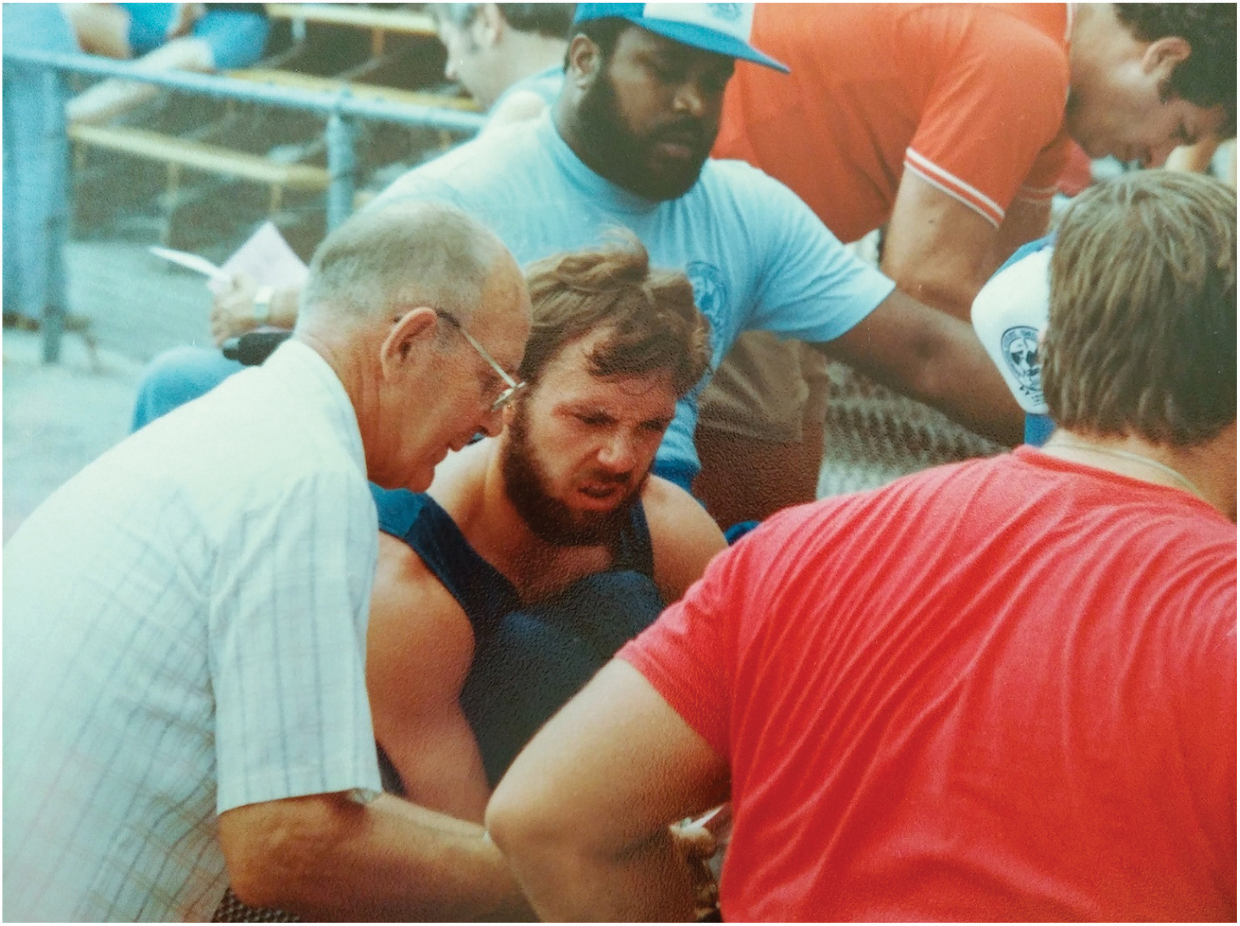
Rory and his grandfather Roy Munn working on Rory’s racing wheelchair.

Cooper’s engineering skills eventually led him to become a professor and researcher at the University of Pittsburgh, where he founded and currently directs the Human Engineering Research Laboratories (HERL). These skills also led to 25 U.S. patents that he currently holds on innovations ranging from advanced wheelchair design and robotic devices to seat cushions and wearable instruments for people with disabilities. His ingenuity also helped him win a bronze medal at the 1988 Paralympic Games (Figure 1). Here is a bit of our conversation.

### RORY COOPER:

**Figure 2.**
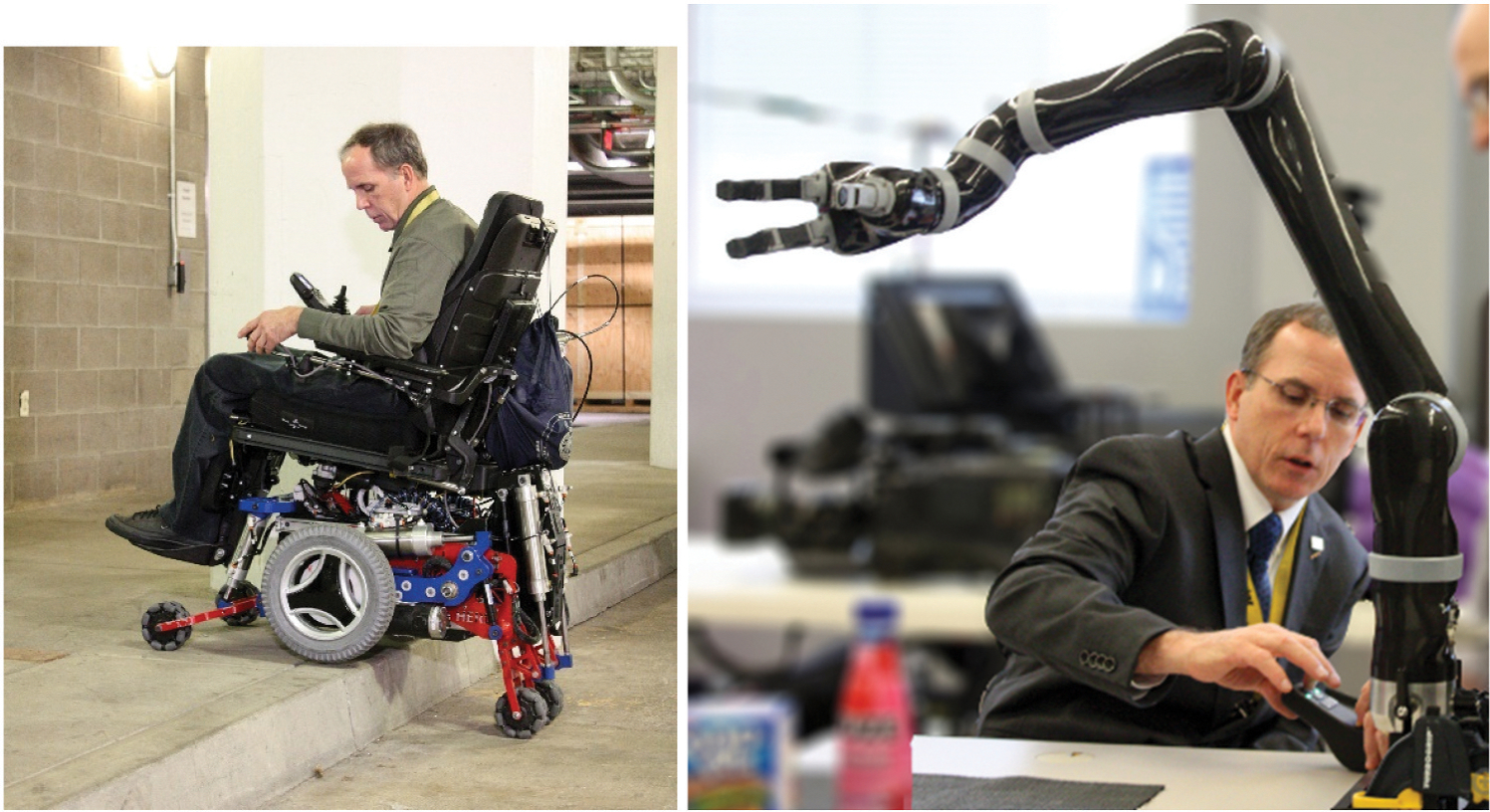
Rory and two of the groundbreaking inventions developed at HERL: at left, the MEBot chair, which can climb stairs, and at right, an “assistive manipulator,” or robotic arm, with an interface that can be controlled by a person’s brain instead of being physically manipulated.

I run a fairly large research group. There’s about 70 of us from the U.S. Department of Veterans Affairs and the University of Pittsburgh. We do research on technology to improve the health and quality of life of people with disabilities and our veterans and service members. We work on wheelchairs, robotic devices, wearable health technologies (Figure 2).

We do some work actually in home modifications or home automation and some work in prosthetics as well. So my patents range from push-ergonomic push rims on wheelchairs that have literally saved the healthcare system billions of dollars, but I also have a patent on a robotic strong arm, which is designed to mount to a person’s power wheelchair to assist with transfers.

And I think [it] was issued this week or last week. . . (LH: Congratulations.) a new patent to . . . thank you . . . for a robotic wheelchair robotic bed to do no-lift transfers in and out of bed. So this is one of the main reasons we have to put people in long-term care facilities or nursing homes, is that a family member or a caregiver cannot help get people in and out of bed anymore, [and it] causes a lot of [lower] back pain among nurses as well. So we invented a device that does this basically controlled with a tablet, [so] that the wheelchair and the bed motions are coordinated. So you basically kind of get poured into bed by the wheelchair, and pulled into bed by the bed, and vice versa when you’re getting out. So, it can all be done with no-lift.

### LINDA HOSLER:

You mentioned the Human Engineering Research Labs. Can you tell me how long you’ve been there and how you shaped it?

### RORY COOPER:

I created it. (LH: Yeah, well, how long ago was that?) So, that was, I guess, probably my biggest invention. It didn’t exist when I was recruited by the VA in Pittsburgh and the University of Pittsburgh. I think we also kind of pioneered the idea of truly multidisciplinary teams. We’re very tied in closely to the UPMC, University of Pittsburgh Medical Center, Center for Assistive Technology, which is run by my wife, Rosi, who is a physical therapist. They see about 3,000 clients a year, so they give us a lot of ideas on what research we should be working on in HERL. So we’re connected to the veterans community, and we’re connected to the active duty military community through Walter Reed and the Center for the Intrepid.

### LINDA HOSLER:

And how long have you been involved in innovative efforts that help veterans specifically?

### RORY COOPER:

Since I was injured. When I got my first wheelchair, it was this 80-pound behemoth that I could hardly get in the car. I got out of the hospital on my birthday in November of 1980, and I knew I wanted to go to college starting in January. So, I started pushing that chair about a mile a day to try to get ready for college. About six weeks later it was broken. Friends of mine in the Army and in Germany did a “fun run” and raised money to buy me my first sort of ultralight wheelchair – at that time they called them sports wheelchairs. It was better, but then I could see there was a lot of room for improvement.

When I was in the hospital, they gave me a back brace for my spinal cord injury, and you couldn’t really sit up with it, and you couldn’t transfer with it. And the doctor, [it] took him a while to figure out that every time I got a pass to go home that brace got different; I was actually modifying the brace when I was at home so it would work for me. Then I started with making better everyday chairs for me. I was lucky, a friend of mine was building a home-built aircraft, so I learned a lot about Kevlar and carbon fibers and using foam. I was probably the first one in wheelchairs to start using composites.

### LINDA HOSLER:

Were you ever a tinkerer before that, or was that really your sort of genesis as an inventor?

### RORY COOPER:

No, I was. I started as a kid. I was lucky my uncle, my father’s brother, was also an engineer. I even made my own mono-shock bicycle in the early 1970s by taking . . . basically, a worn out motorcycle shock and then cutting up one of those Schwinn Stingrays. Of course, at that time I was like 13 years old; I didn’t know about patents. [LH: Yeah.]

But no, I was a tinkerer. And the Army actually even took advantage of that. When I first joined the Army, I was trained as an armorer. . . The Army had me like fix vehicles and stuff like that. And even then, you know, a lot of times if you’ve watched [shows like MASH], a lot of times you don’t have all the parts you need. So, you figure if you can weld a little bit, machine a little bit, you could find a solution.

I was lucky, my mom is a, you know, not a formal inventor, and my grandfather was a railroad machinist and worked for Howard Hughes for a while and helped work on the Spruce Goose and some of the other airplanes. So there’s a lot of times with my mom and my grandfather and I, we were coming up with new wheelchair designs, and my grandfather was supportive enough to follow my crazy ideas when I said I need a tube this size, bed like this. So just a lot of things weren’t available back then, to get through life.

### LINDA HOSLER:

Would you have called yourself an inventor, an engineer, when you were growing up, or did you ever aspire to be one?

### RORY COOPER:

You know, I wasn’t sure what I was going to do. That was the whole point of joining the Army, was to kind of get some new experiences . . . What really convinced me to become an engineer was the Army. But the Army thought I was a better talker than a shooter and trained me to become a civil affairs person and assigned me to a unit called Fifth Signal Command, which does signal communication. So I got to run with a lot of these officers, and they’re eventually the ones that said, “You know, you’re really creative, and you’re kind of smart.” They said, “You should study engineering.” So, when I was injured, I followed their advice and studied engineering.

### LINDA HOSLER:

You talked about wheelchair sports as well and being a competitor.

### RORY COOPER:

A lot of people that are veterans are competitive too, right? [The] military is kind of a competitive environment, and I liked to compete in athletics. So, it was kind of a natural, [that] after I got injured, I would switch to wheelchair sports.

**Figure 3.**
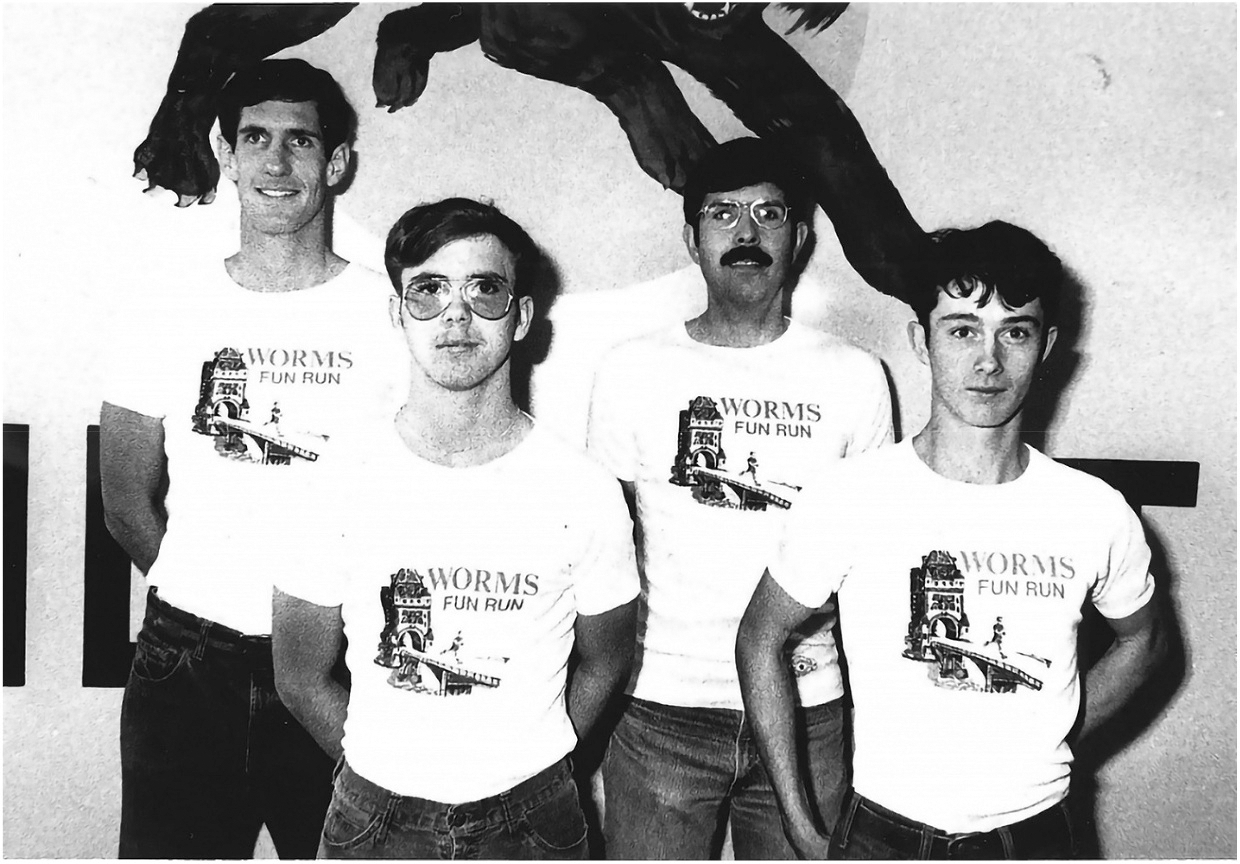
Rory Cooper (front left) and the Army distance running team in 1978, two years before Rory’s accident.

I competed on the U.S. Army Europe track and cross country team when I was a soldier as well, and I competed in track and cross country in high school (Figure 3). I even wrestled for a little bit in high school. You know, my wife will tell you that I’m probably a better engineer than an athlete, and that helped me with my athletic competition. She calls it techno-doping—[using] robots.

### LINDA HOSLER:

In just a sentence or two, could you tell me why accessibility is so important?

### RORY COOPER:

**Figure 4.**
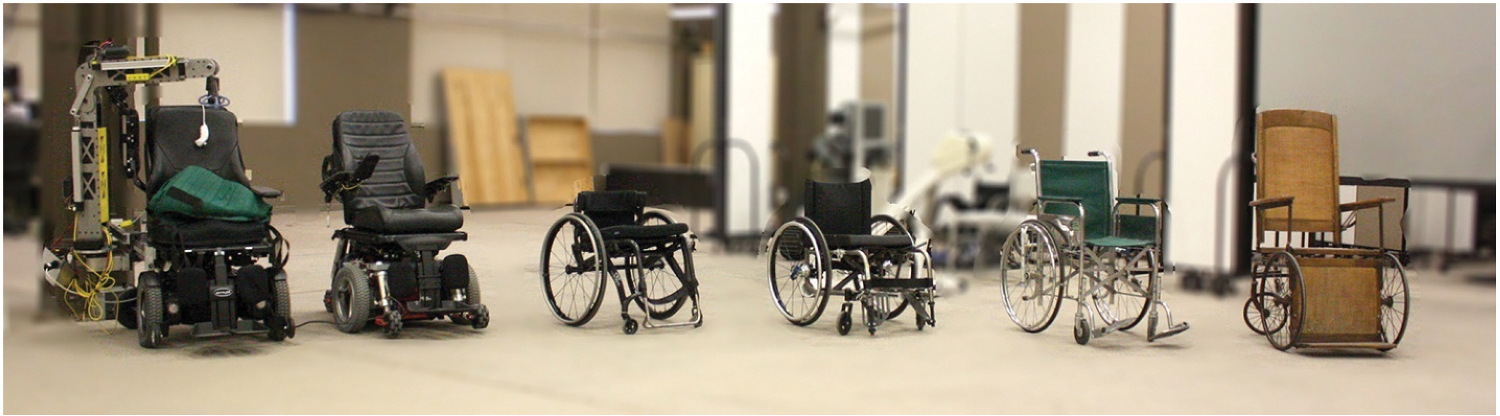
The evolution of wheelchairs, right to left: a 19th-century wheelchair, a hospital-style wheelchair circa 1935–1975, a folding manual wheelchair, an ultralight manual wheelchair, a MEBot chair that can navigate curbs and other obstacles, and a strong arm robotic transfer device on a power chair, developed at HERL.

Accessibility helps drive social inclusion. If you think about it, the wheelchair I use, the adaptive vehicle I use, the home modifications I use, they all allow me to be productive and creative and contribute to society. What’s important is to create a world where everyone belongs and everyone can contribute. Accessibility is a tool to facilitate that (Figure 4).

### LINDA HOSLER:

One thing that I hear a lot from inventors is how failure is a part of invention. Do you have a moment where you thought you failed and you wanted to give up but you didn’t, for whatever reason – or how you get past feeling like something had failed?

### RORY COOPER:

It happens to . . . us all the time. We have a pretty good success rate, and it’s probably on the order of 50, 60%. And that’s a good rate, right? That’s among the best. In failing, when you’re building something, if you’re trying to create something that’s never existed before, I don’t really consider that failing. You learn something all the time. We just have to find maybe another approach to solving that problem.

### LINDA HOSLER:

Speaking to a wide audience, what advice would you give to other innovators?

### RORY COOPER:

Keep learning, and learn from all kinds of people – other engineers, from scientists [and] other disciplines. I mean, a lot of invention is really taking bits and pieces. You’re seeing a problem, learning how to translate that problem into a potential solution or pathway to a solution. And then being able to integrate a lot of different knowledge from different disciplines. And don’t be afraid to talk to other people.

You know when I was first injured, I was told by the doctors that, you know, you’d probably have a 10-year lifespan, right? And I could’ve just said, well then I’ll just hang out and do nothing and wait for my time to come instead of trying to do as much with my life as I could. But also, you know, you get people saying, well, you really can’t be an engineer because you have to do all this physical and mental work. I was able to convince professors that, really, that should be an engineering problem. How do you take a person with a disability and help them learn engineering?

### LINDA HOSLER:

And my last question for you is, looking back at the impacts of all your inventions, what are you most proud of?

### RORY COOPER:

There’s really two answers to that. So, the first is the people that I’ve inspired to go on and be inventors and start companies or go to school and be an engineer. Many of those are themselves veterans or people with disabilities . . . or have a family member that’s been affected and want to now make a difference. I think the other one is, for me, that’s really fun, is I can literally go anywhere in the world and see somebody with one of my inventions.

One of my favorite things to do is go up to somebody I don’t know [when] I see them using one of my inventions. I kind of ask them about it: What did they think? How does it help? And when people say, “Oh, it’s wonderful, it’s transformed my life, and I don’t have any pain anymore” or “I just don’t have pressure ulcers now” or “I used to be propelled by my mom, and now I can drive my chair by myself,” I tell them I helped invent that device. And it’s always cool to see the reaction on their face. A lot of people, go, “Oh, can I get a picture with you, and what’s your name?” And . . . some people now will actually go online immediately and say, wow, that’s really you. And that’s kind of cool to see when you get to meet people and see how it’s affected their lives.

### LINDA HOSLER:

**Figure 5.**
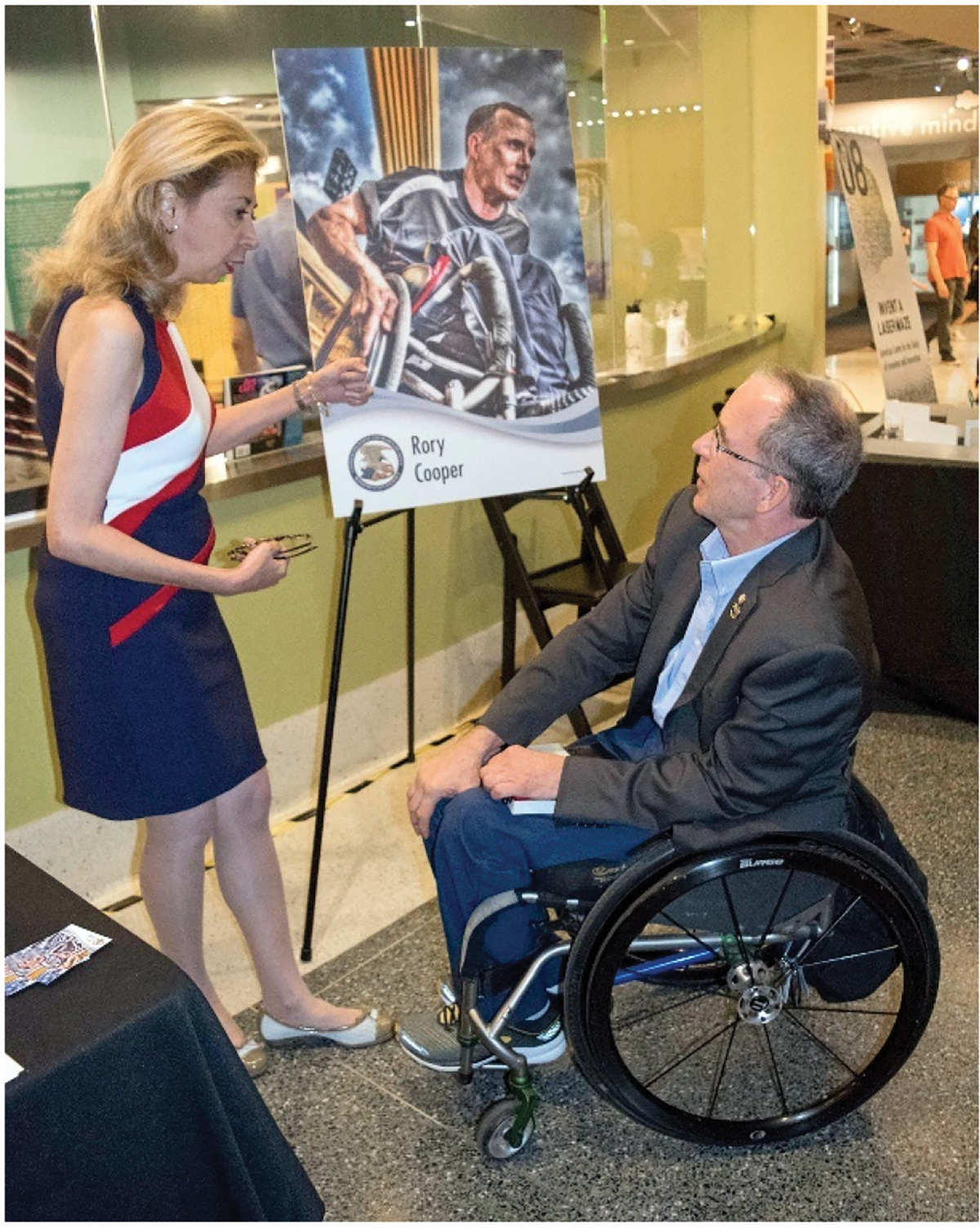
Rory and deputy under secretary of commerce for intellectual property and deputy director of the USPTO Laura Peter at the Smithsonian’s military invention Day 2019, with a display of the USPTO’s inventor trading card depicting Cooper and some of his patented inventions in the field of wheelchairs and robotics.

Now, four decades and 25 patents after his accident, Rory Cooper has gotten to see the impact of his work all over the world. We thank him for taking the time to give us insight on his inspiring journey and for discussing his groundbreaking work – for making life after injury not just bearable, but better, and for making the world a more accessible place. From the USPTO, thanks for listening (Figure 5).

